# Emerging Evidence Highlighting the Importance of Redox Dysregulation in the Pathogenesis of Amyotrophic Lateral Sclerosis (ALS)

**DOI:** 10.3389/fncel.2020.581950

**Published:** 2021-02-18

**Authors:** Cyril Jones Jagaraj, Sonam Parakh, Julie D. Atkin

**Affiliations:** ^1^Department of Biomedical Sciences, Macquarie University Centre for MND Research, Faculty of Medicine and Health Sciences, Macquarie University, Sydney, NSW, Australia; ^2^Department of Biochemistry and Genetics, La Trobe Institute for Molecular Science, La Trobe University, Bundoora, VIC, Australia

**Keywords:** redox dysregulation, ALS pathogenesis, oxidative stress, PDI—protein disulfide isomerase, SOD1, ROS—reactive oxygen species

## Abstract

The cellular redox state, or balance between cellular oxidation and reduction reactions, serves as a vital antioxidant defence system that is linked to all important cellular activities. Redox regulation is therefore a fundamental cellular process for aerobic organisms. Whilst oxidative stress is well described in neurodegenerative disorders including amyotrophic lateral sclerosis (ALS), other aspects of redox dysfunction and their contributions to pathophysiology are only just emerging. ALS is a fatal neurodegenerative disease affecting motor neurons, with few useful treatments. Hence there is an urgent need to develop more effective therapeutics in the future. Here, we discuss the increasing evidence for redox dysregulation as an important and primary contributor to ALS pathogenesis, which is associated with multiple disease mechanisms. Understanding the connection between redox homeostasis, proteins that mediate redox regulation, and disease pathophysiology in ALS, may facilitate a better understanding of disease mechanisms, and lead to the design of better therapeutic strategies.

## Introduction

Amyotrophic lateral sclerosis (ALS), or motor neuron disease (MND), is a fatal neurodegenerative disorder associated with aging, with an average survival time of 2–5 years following diagnosis. ALS is characterized by the degeneration of both upper motor neurons, which project from the cortex to the brainstem and the spinal cord, and lower motor neurons, which project from the brainstem or spinal cord to muscle. ALS commonly begins in late adulthood and many patients present with spinal onset ALS, characterized by muscle weakness in the limbs. However, in others, ALS begins in the bulbar muscles, which is characterized by difficulties in speech and swallowing. Patients develop progressive paralysis and the disease advances rapidly until at end-stage, only support and palliation are available. Death usually occurs from respiratory failure. ALS also shares neuropathological similarities with frontotemporal dementia (FTD). In fact, ALS and FTD represent two opposite ends of the same disease spectrum, with overlapping clinical symptoms and genetics (Shahheydari et al., 2017).

The cellular redox state, or balance between cellular oxidation/reduction reactions, serves as a vital antioxidant defence system that is linked to all important cellular activities (Calabrese et al., 2010). Redox regulation is, therefore, a fundamental cellular process for aerobic organisms. The imbalance between the production and accumulation of reactive species in cells leads to oxidative and nitrosative stress, producing reactive oxygen species (ROS) and reactive nitrogen species (RNS). Dysregulation of redox conditions alters the cellular redox state, modifies redox-sensitive proteins, and disrupts redox-regulated mechanisms.

Redox homeostasis controls multiple cellular signaling pathways including proper functioning of the mitochondria and endoplasmic reticulum (ER) compartments, calcium regulation, axonal transport, autophagy, protein folding, and proteostasis (Görlach et al., 2006; Cao and Kaufman, 2014; Li P. et al., 2016; Yoboue et al., 2018; Guerrero-Gómez et al., 2019). Not surprisingly, dysregulation in the cellular redox state is implicated in many diseases, including ALS (Parakh et al., 2013; Mcbean et al., 2015; Pinho et al., 2020). Furthermore, during the normal aging processes, the ability of cells to maintain their normal redox state diminishes (Go and Jones, 2017; Castelli et al., 2019). Whilst this is true of all cell types, neurons are particularly susceptible to redox dysregulation due to their large size and high consumption of oxygen. Furthermore, they produce significant quantities of ROS and RNS (Kato et al., 2005). Moreover, disruption to redox dysregulation is increasingly implicated as an important driver of neurodegeneration in the pathogenesis of many age-related neurodegenerative disorders, including ALS. Also, several pathogenic mechanisms linked to ALS involve redox-sensitive proteins, including protein disulfide isomerase (PDI), thioredoxin, and glutathione (GSH), and recent evidence highlights their importance in neurodegeneration. However, whilst redox dysfunction has been associated with ALS for some time (Harraz et al., 2008; Cohen et al., 2012; Conrad et al., 2013; Parakh et al., 2020), a precise understanding of how cellular redox conditions are dysregulated has been lacking. Nevertheless, recent evidence implies that redox homeostasis is a central and primary mechanism in ALS and it may have greater importance than previously recognized (Sbodio et al., 2019; Parakh et al., 2020). Here, we provide a comprehensive review of the evidence linking redox dysfunction to ALS. We discuss the cellular redox system, the major pathological proteins and pathways associated with ALS, and their relationship to redox homeostasis. We also describe how redox modifications are associated with ALS-like phenotypes.

## Amyotrophic Lateral Sclerosis (Als)

ALS is a multifactorial neurodegenerative disease (Taylor et al., 2016). Most cases (~90%) have no previous family history and hence are termed “sporadic ALS (sALS).” Specific environmental factors may increase the risk of developing ALS, including smoking, air pollution, agricultural and industrial pollutants, β-N-methyl amino-L-alanine (BMAA) toxicity, and physical activity. However, previous studies have yielded conflicting results, so the role of environmental factors involved in triggering ALS remains unclear (Bozzoni et al., 2016; Wood, 2016; Yu and Pamphlett, 2017). Genetic mutations account for the remaining approximately 10% of cases, termed “familial” ALS (fALS; Taylor et al., 2016). ALS shares clinical and pathological attributes with FTD, which is characterized by deterioration in behavior, personality, and/or language. In FTD, neurons in the frontal and temporal lobes of the brain primarily degenerate and die (Neumann et al., 2006; Burrell et al., 2016). ALS and FTD are closely related genetically and pathologically, and these two conditions are now considered to be at opposite ends of the same disease continuum (Shahheydari et al., 2017). Like other neurodegenerative diseases, ALS is a protein misfolding disorder and abnormal misfolded protein inclusions are present in motor neurons and glia.

Most familial cases of both ALS (~40%) and FTD (25%) are associated with hexanucleotide (G4C2) expansions in the *Chromosome 9 open reading frame 72* *(C9orf72)* gene (Dejesus-Hernandez et al., 2011; Renton et al., 2011; Bigio, 2012; Lee Y.-B. et al., 2013; Ling et al., 2013; Balendra and Isaacs, 2018), followed by mutations in *superoxide dismutase 1* (*SOD1*; 20%), *TARDBP* (4–5%), encoding TAR DNA-Binding Protein-43 (TDP-43), and *Fused in Sarcoma* (*FUS*; 5%). Interestingly, genetic mutations in sALS patients have also been described, in *C9orf72* (7% of cases), *TARDBP, SOD1, FUS, VCP, p62, PFN-1*, MATR3, *OPTN, UBQLN2*, *CHCHD10, TBK1*, *TUBA4A, NEK1, C21orf2*, and *CCNF*, together accounting for 15% of sporadic ALS cases (Taylor et al., 2016; Chia et al., 2018). Table 1 summarizes the genetic mutations identified in both familial and sporadic ALS patients.

**Table 1:** Genetic mutations identified in familial and sporadic forms of amyotrophic lateral sclerosis (ALS).

Gene	Locus	Protein encoded	sALS (%) Frequency	fALS (%) Frequency	Cellular function	References
*SOD1*	21q22.1	Superoxide dismutase 1	1–2	12–23.5	Redox regulation and antioxidant activity	Rosen et al. (1993) and Andersen et al. (2003)
*DAO*	12q22-23	D-amino acid oxidase	NA	Unknown	Enzyme that catalyzes the oxidative deamination of D-amino acids	Mitchell et al. (2010)
*C9orf72*	9p21.2	Chromosome 9 open reading frame72	7	30–50	Rab mediated cellular trafficking, autophagy, autoimmunity, indirect oxidative stress, and vesicular trafficking	Dejesus-Hernandez et al. (2011) and Renton et al. (2011)
TARDBP	1p36.22	TAR DNA-binding protein-43	1	2–5	RNA metabolism, DNA repair	Rutherford et al. (2008), Sreedharan et al. (2008), and Kirby et al. (2010)
*FUS*	16p11.2	Fused in Sarcoma	1	5	RNA metabolism, DNA repair	Kwiatkowski et al. (2009) and Vance et al. (2009)
*VCP*	9p13.3	Valosin-containing protein	1	<1	Protein degradation, intracellular membrane fusion and protein quality control	Shaw (2010)
*TBK1*	12q14.2	Tank-binding kinase1	<1	<1–5	Kinase involved in autophagy, inflammation	Cirulli et al. (2015), Freischmidt et al. (2015) and Gijselinck et al. (2015)
*CHCHD10*	22q11.23	Coiled-coil-helix-coiled-coil-helix domain containing 10	<1	3.6	Mitochondrial regulation	Bannwarth et al. (2014) and Zhang et al. (2015)
*SQSTM1/p62*	5q35	Sequestosome 1/p62	<1	1.8	Autophagy receptor involved in selective autophagy, UPS	Fecto et al. (2011) and Le Ber et al. (2013)
*CCNF*	16p13.3	Cyclin F	2	0.6–3.3	Regulator of cell cyclin transitions, a component of the E3 Ubiquitin protein ligase linked to protein homeostasis, UPS	Williams et al. (2016)
*PFN1*	17p13	Profilin-1	<1	<1	Actin binding protein involved in regulating actin dynamics	Wu et al. (2012)
*ALS2*	2q33	Alsin	NA	Unknown	Cellular trafficking, neuroinflammation, redox regulation	Yang et al. (2001)
*KIF5A*	12q13.3	Kinesin family of proteins	NA	<1	Molecular motor protein involved in axonal transport	Brenner et al. (2018) and Nicolas et al. (2018)
*OPTN*	10p13	Optineurin	<1	2.6	Maintenance of Golgi complex, membrane trafficking, and autophagy receptor	Maruyama et al. (2010) and Pottier et al. (2015)
*UBQLN2*	Xp11.21	Ubiquilin 2	<1	0.5–2	Macroautophagy and the UPS	Deng et al. (2011), Synofzik et al. (2012), Williams et al. (2012), and Gellera et al. (2013)
*NEFH*	22q12	Neurofilament heavy chain		Rare	Component of the cytoskeleton that provides structural support and facilitates axonal transport	Figlewicz et al. (1994)
*TUBA4A*	2q36.1	Tubulin α-4A chain	<1	<1	Microtubule subunit involved in intracellular transport and DNA segregation	Smith et al. (2014)

Mutations in SOD1, TDP-43, C9orf72, and FUS therefore together account for a large proportion of fALS cases and have been extensively studied *in vitro* (Taylor et al., 2016). Furthermore, disease models based on the transgenic expression of ALS-associated mutations *in vivo* have been used extensively to investigate ALS pathogenesis and motor neuron degeneration (Taylor et al., 2016). From these studies, many pathogenic mechanisms have been described, including dysfunction to redox homeostasis (Taylor et al., 2016; Hardiman et al., 2017; Mejzini et al., 2019). Transgenic mice expressing human SOD1 mutants, particularly SOD1^G93A^, are the most extensively studied animal used for ALS.

Most of the ALS mutations are point mutations or truncations. However, C9orf72 contains a hexanucleotide repeat expansion, hence it is distinct from the other ALS/FTD mutations. Both losses of normal cellular function and gain of aberrant toxic functions (or both together) are implicated as pathogenic mechanisms in ALS, depending on the protein involved. Both loss and gain of functions have been proposed for mutant TDP-43 and mutant FUS, whereas gain of a toxic function is the preferred mechanism associated with mutant SOD1. Interestingly, two possible gain of functions mechanisms are thought to be induced by the C9orf72 repeat expansion; toxicity from the long repeat RNA or the production of dipeptide repeat proteins (DPR). These DPRs are generated by repeat-associated non-ATG translation (RAN translation), and five distinct peptides are produced (GA, GP, GR, PR, and PA) resulting from translation of both sense and antisense strands (Dejesus-Hernandez et al., 2011; Renton et al., 2011). Haploinsufficiency, due to reduced expression of C9orf72 in ALS patients, is also implicated in pathogenesis (Sellier et al., 2016).

Interestingly, the proteins encoded by these genes display diverse functions. The normal cellular function of C9orf72 is related to vesicular trafficking and autophagy (Farg et al., 2014) whereas SOD1 is an antioxidant enzyme that mediates detoxification of the superoxide anion radical ($O_{\text{2}}^{-}$; Mccord and Fridovich, 1969). In contrast, both TDP-43 and FUS are RNA and DNA binding proteins found predominantly in the nucleus, where they regulate transcription, the DNA response, mRNA splicing, RNA stability, micro-RNA biogenesis, translation and transport, and stress granule formation (Cohen et al., 2011; Loughlin and Wilce, 2019; Birsa et al., 2020). TDP-43 is an important pathological protein because the abnormal accumulation of misfolded, wildtype (WT) cytoplasmic TDP-43 into inclusions is the characteristic pathological hallmark of ~97% of ALS patients (sALS and fALS; Neumann et al., 2006). Similarly, mislocalization of WT, misfolded FUS into the cytoplasm was recently implicated as a key pathological hallmark of ALS (Neumann et al., 2006; Tyzack et al., 2019). TDP-43 and FUS, therefore, share strong structural, functional, and pathological similarities, distinct from C9orf72 and SOD1. However, as well as TDP-43 and FUS, WT SOD1 has also been described in the inclusions in sporadic ALS motor neurons (Tokuda et al., 2019).

### Disease Mechanisms in ALS

Whilst the proteins associated genetically or pathologically with ALS are diverse in function and structure, similar disease mechanisms are implicated. Moreover, there are a plethora of different processes known to be dysregulated in ALS. However, ultimately it is imperative to elucidate the primary, upstream mechanisms responsible for neurodegeneration in ALS so that effective therapeutics can be designed. These mechanisms include impaired axonal transport (Collard et al., 1995; Williamson and Cleveland, 1999), neurofilament aggregation (Al-Chalabi et al., 1995; Xiao et al., 2006; Xu Z. et al., 2016), protein misfolding (Kopito, 2000; Basso et al., 2006), abnormal RNA processing (Chen et al., 2010; Dejesus-Hernandez et al., 2011; Parisi et al., 2013; Droppelmann et al., 2014), lipid peroxidation (Shibata et al., 2001) and cholesterol esterification (Cutler et al., 2002; Chaves-Filho et al., 2019), defects in nucleocytoplasmic transport (Boeynaems et al., 2016), induction of DNA damage (Konopka and Atkin, 2018; Naumann et al., 2018; Konopka et al., 2020), cytoplasmic mislocalization of nuclear proteins (Neumann et al., 2006), mitochondrial dysfunction (Albers and Beal, 2000), glutamate excitotoxicity (Shaw and Ince, 1997), proteasomal and autophagic dysfunction (Chen et al., 2012), ER stress (Nagata et al., 2007; Walker et al., 2010), mitochondrial associated membrane (MAM) dysfunction (Watanabe et al., 2016), ER-Golgi transport defects (Atkin et al., 2014; Soo et al., 2015), autophagy dysregulation and apoptosis (Ravits et al., 2013; Robberecht and Philips, 2013; Gao et al., 2017; Mandrioli et al., 2020). For a detailed discussion of these mechanisms, please see several excellent reviews (Zarei et al., 2015; Taylor et al., 2016; Weishaupt et al., 2016; Mejzini et al., 2019). Here we will focus only on those mechanisms linked to cellular redox processes.

Mechanisms of redox regulation are a vital antioxidant defense system that underpins many important cellular activities. Not surprisingly, redox dysfunction is implicated as an important pathogenic mechanism in ALS. Mutations in many ALS-associated genes are associated with cellular redox dysregulation, particularly *SOD1*, *C9orf72, FUS*, *TDP-43*, *CHCHD10*, and *ALS2*. Furthermore, mutations in other ALS genes disrupt the cellular redox balance and induce oxidative stress (Carter et al., 2009). Also, mutations in genes encoding proteins that are involved in maintaining redox homeostasis are present in ALS patients, such as SOD1 and D-amino acid oxidase (DAO; Mitchell et al., 2010; Kondori et al., 2018). Moreover, dysregulation of redox homeostasis is also present in sALS patient tissues (Kato et al., 2005), thus placing redox dysregulation onto the pathophysiology of the most common forms of ALS. The fundamental processes underlying control of the cellular redox environment will now be discussed.

## The Cellular Redox System

The cellular redox state refers to the balance between oxidation and reduction reactions (Calabrese et al., 2010; Sies et al., 2017; Sies and Jones, 2020). Redox regulation involves proteins and cofactors that maintain the appropriate redox environment for proper functioning of the cell (Ray et al., 2012), ensuring that there is a balance between the production of ROS, RNS, and their consequent elimination by antioxidant enzymes and smaller molecules. Cells have developed a sophisticated antioxidant system to protect against these oxidative insults, consisting of enzymes that either convert superoxide radicals into hydrogen peroxide (H_2_O_2_, SOD1, and catalases) or H_2_O_2_ into water and oxygen [peroxiredoxin (Prx) and glutathione peroxidase (GPx); Fridovich, 1986; Espinosa-Diez et al., 2015]. Prx and GPx require secondary enzymes and cofactors to function efficiently and regulate the cytoplasmic redox system (Chae et al., 1994).

The overall cellular redox state is determined by two cellular disulfide reductase systems (Das and White, 2002; Ren et al., 2017). First, the thioredoxin system comprises thioredoxin (Trx), thioredoxin reductase (TrxR), thioredoxin peroxidase (TrxP), and nicotinamide adenine dinucleotide phosphate (NADPH; Lee S. et al., 2013; Lu and Holmgren, 2013). Trxs are ubiquitous antioxidant enzymes containing thiol groups (-SH) that are present in cysteine residues (Collet and Messens, 2010). Thiol groups are important components of redox-mediated processes due to their unique chemistry, involving nucleophilicity, metal binding, and the ability to form protein disulfide bonds (Ulrich and Jakob, 2019). Hence, whilst cysteine is one of the less common amino acids, it is often highly conserved within protein functional groups (Marino and Gladyshev, 2010). Second, the glutathione system comprises NADPH, glutathione reductase (GR), glutathione peroxidase (GPx), and glutathione (GSH; Ursini et al., 2016; Ren et al., 2017). GSH is a tripeptide consisting of cysteine, glutamic acid, and glycine, and it is an important cellular antioxidant. In fact, it is the most abundant low molecular weight thiol-containing compound produced in cells. NADPH is the principal reductant used to maintain the redox states of both the Trx and GSH systems. Trx and GSH control redox signaling and regulate cellular H_2_O_2_ concentrations through Prxs and GPxs. Trx and GSH are found in several different subcellular compartments, including the nucleus, cytoplasm and mitochondria, in both neuronal and glial cells (Ren et al., 2017).

**Figure 1:**
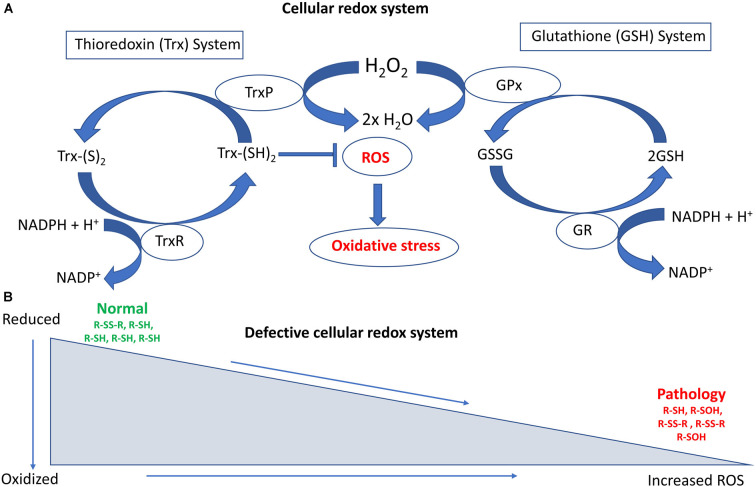
Schematic diagram illustrating the major cellular redox systems.** (A)** Cellular redox processes include the thioredoxin (Trx) and glutathione (GSH) systems that reversibly regulate thiol modifications. The overall redox state of the cell is determined by these two cellular disulfide reductase systems. The thioredoxin system comprises thioredoxin reductase (TrxR), nicotinamide adenine dinucleotide phosphate (NAPH), thioredoxin peroxidase (TrxP), and Trx. TrxR is involved in the conversion of Trx-(S)_2_ (oxidized form of Trx) into Trx-(SH)_2_ (reduced form of Trx), whereas TrxP is involved in the conversion of Trx (SH)_2_ into Trx-(S)_2_. The glutathione system comprises GR (Glutathione reductase), NADPH, GSH, and GPx (Glutathione peroxidase). GR is involved in converting GSSG (Glutathione disulfide) to GSH whereas GPx is involved in converting GSH to GSSH. Overloading of the Trx system can increase reactive oxygen species (ROS) accumulation and oxidative stress. **(B)** Defective cellular redox conditions can produce increased levels of ROS. This can lead to irreversible thiol modifications such as sulfinic or sulfonic acid (−SO_3_H) formation, as well as induce protein degradation and associated pathological events in amyotrophic lateral sclerosis (ALS).

The reduced form of glutathione (reduced GSH) is the biologically active species that regulates the cellular antioxidant defense system. GSH also maintains the intracellular redox milieu to preserve the thiol-disulfide redox states of proteins (Morgan et al., 2013). Interestingly, the proper folding of proteins and the formation of protein disulfide bonds both depend on the redox status within the ER. Importantly, the lumen of the ER represents a more oxidizing environment than the cytoplasm, with a higher ratio of oxidized to reduced glutathione (GSSG/GSH; Van Der Vlies et al., 2003). In the ER, the oxidoreductase enzymes endoplasmic oxidoreductin 1 (Ero1) and PDI facilitate disulfide bond formation in substrate proteins. Redox dysfunction can affect their activity, and in particular reduced GSH or protein thiols react with and generate ROS, inducing ER stress (Hatahet and Ruddock, 2009; Ramming and Appenzeller-Herzog, 2012), which dysregulates cellular redox homeostasis (Espinosa-Diez et al., 2015). Figure 1 summarizes the major cellular redox systems.

## Redox Dysregulation

Oxidative stress results from elevated intracellular levels of ROS, and nitrosative stress results from increased levels of RNS, and both events can significantly damage cells. Even slight modulations in the cellular redox state can produce neurotoxic species. ROS includes free radicals (superoxide ($O_{\text{2}}^{-}$) and hydroxyl radicals (^.^OH), whereas RNS includes nitric oxide (NO) and nitrogen dioxide (NO_2_; Valko et al., 2007). Redox homeostasis ensures that cells respond to these redox stressors efficiently. However, when it is disturbed, neurodegeneration can result (Parakh et al., 2013; Mcbean et al., 2015). The primary sources of ROS production in the central nervous system (CNS) are mitochondrial proteins, NADPH oxidase, Rac1, and SOD1 (Nayernia et al., 2014; Di Meo et al., 2016). The generation of ROS can be activated by endogenous factors, such as the mitochondrial electron transport chain (ETC), NADPH oxidases (NOX), lipoxygenases (LOX), cytochrome P450, and xanthine oxidase (XO), or by exogenous causes such as pollutants, chemicals/drugs, radiation and heavy metals (Moussa et al., 2019). Oxidation of GSH to GSSG results in dysregulation of the intracellular redox imbalance (decreased GSH:GSSG ratio), which is associated with oxidative stress and DNA damage in fibroblasts (Asensi et al., 1999).

Modification of cysteine thiols by ROS and RNS has emerged as an important mechanism of altering protein structure and function (Cai and Yan, 2013). Many proteins undergo reversible thiol modifications during physiological redox signaling processes as part of cellular defense mechanisms against oxidative and nitrosative damage (Brandes et al., 2009). There is a broad range of possible alterations to these thiols, including S-nitrosylation (-SNO), S-sulfenylation (sulfenic acid, -SOH), S-glutathionylation (-SSG), disulfide formation (-S-S-), S-sulfhydration (−S-SH), and S-sulfinylation (−SO_2_H; Hawkins et al., 2009; Mieyal and Chock, 2012; Finelli, 2020). However, in addition to these reversible modifications, several cysteine adducts can form irreversibly due to prolonged exposure of cysteine residues to ROS and RNS, such as sulfinic or sulfonic acids (Chouchani et al., 2011; Paulsen and Carroll, 2013). These irreversible thiol modifications can lead to protein degradation and loss of function and are present in neurodegenerative diseases (Ren et al., 2017).

## Direct Evidence for A Role of Redox Dysregulation in Als

In this section, we discuss evidence demonstrating that the central cellular redox regulatory mechanisms and associated proteins are perturbed in ALS (Table 2). This includes NOX, apurinic/apyrimidinic endonuclease 1 (APE1), Prx, thioredoxin (TRX)-related transmembrane-2 (TMX2), activator protein 1 (AP-1), PDI, and SOD1. Later we discuss more indirect evidence for dysregulation to redox homeostasis in ALS.

**Table 2:** Redox related proteins associated dysregulation in ALS.

Protein Name	Function	Abnormalities in ALS	References
SOD1	This cytosolic enzyme facilitates the conversion of the superoxide anion to hydrogen peroxide and oxygen.	Mutant SOD1 disrupts redox homeostasis in ALS by abnormal production of ROS and RNS, and by the formation of misfolded protein aggregates.	Mccord and Fridovich (1969), Bruijn et al. (2004), and Wu et al. (2006)
NOX	NOX produces the substrate superoxide anion for the reaction catalyzed by SOD1 and controls the production of pro-inflammatory cytokines.	Elevated levels of NOX2, $O_{\text{2}}^{-}$ and inactivation of NOX1 were observed in SOD1^G93A^ mice. Deletion of NOX improves the survival of SOD1^G93A^ mice. Mutant SOD1 alters NOX-dependent redox stress.	Mccord and Fridovich (1969) and Dunckley et al. (2007)
APE1	Regulates multiple transcription factors; NF-κB, STAT3, AP-1, HIF-1, and p53. APE1 interacts with proteins involved in both DNA repair and redox regulation.	APE1 is upregulated in motor neurons, astrocytes, and spinal cords of ALS patients. Mutant SOD1^G93A^ restricts the localization of APE1 and inhibits redox homeostasis.	Shaikh and Martin (2002) and Kim et al. (2020)
Peroxiredoxins	Group of antioxidant enzymes that regulate peroxide and peroxynitrite.	Prx 3 is downregulated in SOD1^G93A^ mice, SOD1, and SALS patients. Prx1, 2, and 6 were upregulated in SOD1^G93A^ mice. Prx3 and Prx5 were found in mutant SOD1 aggregates.	Kato et al. (2005), Wood-Allum et al. (2006), and Knoops et al. (2016)
TMX2	Important sensor required for the maintenance of cellular redox homeostasis.	TMX2 is a protective modifier against C9orf72 DPR toxicity. Depletion of TMX2 suppresses PR_20_ induced cellular toxicity.	Kramer et al. (2018)
AP-1	A redox-sensitive transcription factor that regulates gene expression.	Mutant SOD1 upregulates AP-1 and AP-1 complex proteins like JUN and FOSL1.	Gomez Del Arco et al. (1997), Bhinge et al. (2017), and Kale et al. (2018)
PDI	A chaperone involved in protein folding. It also regulates the cellular redox state and signaling.	PDI levels are upregulated in transgenic mouse models of ALS and ALS patients. Mutations in PDI are also associated with the risk of developing ALS. The redox activity of PDI is protective in cellular models and zebrafish.	Walker et al. (2010), Woehlbier et al. (2016), and Parakh et al. (2020)

### Induction of Oxidative Stress

It is well established that oxidative and nitrosative stress markers are upregulated in ALS patients and disease models (Yang et al., 2001; Cereda et al., 2006; Babu et al., 2008; Lee et al., 2009; Cozzolino et al., 2012; D’Amico et al., 2013). Increased ROS, RNS, and products of oxidation, have been observed both in post-mortem human samples and in SOD1^G93A^ mice (Carrí et al., 2003). Oxidative stress has also been linked to the abnormal accumulation of misfolded SOD1 in ALS patients, and in transgenic *C. elegans* expressing mutants SOD1^A4V^, SOD1^G37R^, or SOD1^G93A^ (Oeda et al., 2001). Furthermore, oxidative stress has been extensively studied in cells expressing mutant SOD1 as well as in transgenic SOD1^G93A^ mice, revealing that various SOD1 mutants increase oxidative stress and dysregulate redox homeostasis (Ferri et al., 2006; Marden et al., 2007; Fukai and Ushio-Fukai, 2011).

An ALS clinical trial administering 600 mg of GSH reported a slightly decreased rate of disease progression (Chili et al., 1998). A significantly lower level of GSH was detected in serum of human sALS patients compared to controls (Ehrhart et al., 2015). Furthermore, reduction in the levels of intracellular GSH increases oxidative stress, mitochondrial dysfunction, and apoptosis in SOD1^G93A^ mice models (Chi et al., 2007). Decreased levels of GSH in WT SOD1 expressing mice were associated with fewer motor neurons and a shorter average lifespan (Killoy et al., 2018). Oxidative stress induced by GSH depletion also reproduces pathological features of TDP-43 in neuronal cells; phosphorylation, cytoplasmic re-distribution, and aggregation (Iguchi et al., 2012). The addition of GSH or expression of Grxs 1 and 2 significantly improves mutant SOD1 solubility in cell culture, whereas reducing the levels of intracellular GSH decreases SOD1 solubility, suggesting that GSH reduction promotes the aggregation of mutant SOD1 (Guareschi et al., 2012).

Treating cells with L-buthionine sulfoximine (BSO), an inhibitor that blocks the synthesis of GSH, renders neuronal SH-SY5Y cells expressing mutant valosin-containing protein (VCP) R487H more susceptible to oxidative stress (Hirano et al., 2015) and increases mutant SOD1 toxicity (Alvarez-Zaldiernas et al., 2016; Bakavayev et al., 2019). Furthermore, BSO induces the misfolding of mutant TDP-43 and mutant SOD1 (see “Protein Folding” section). Similarly, expression of mutant TDP-43 in cellular, yeast, and *Drosophila* models increases markers of oxidative stress, including protein carbonylation and glutathione S transferase D1 (Duan et al., 2010; Braun et al., 2011; Zhan et al., 2015). Furthermore, several micro-RNAs known to regulate the expression of genes involved in counteracting ROS/RNS are differentially regulated in ALS patients. Specific micro-RNAs were upregulated; miR-27a, miR-338-3p, miR-155, whereas other micro-RNAs were downregulated in ALS patients; miR-142-5p, and miR-34a (Koval et al., 2013; Waller et al., 2017; Ricci et al., 2018; Li C. et al., 2019). A meta-data analysis of oxidative stress biomarkers from 41 studies involving a total of 4,588 ALS patients and 6,344 control subjects, revealed a significant increase in malondialdehyde, 8-hydroxyguanosine and advanced oxidation protein products in ALS patients compared to controls (Wang Z. et al., 2019). However, the levels of other oxidative stress markers, uric acid, and GSH were significantly reduced in ALS patients (Wang Z. et al., 2019). Furthermore, no significant changes in the levels of other markers were observed; blood Cu, SOD1, glutathione peroxidase, ceruloplasmin, triglycerides, total cholesterol, low-density lipoprotein, high-density lipoprotein, coenzyme-Q10, and transferrin l; (Wang Z. et al., 2019).

There is also evidence that FUS is involved in the cellular response to oxidative DNA damage, and it is well established that FUS has significant functions in DNA repair (Wang et al., 2013; Naumann et al., 2018). Immunoprecipitation studies revealed an increased association of FUS with XRCC1, LigIII, and PARP-1, but not with other base excision repair (BER) proteins in ALS patient-derived iPSC lines carrying FUS mutations (R521H and P525L). This implies the presence of defects in DNA nick ligation and oxidative damage, and DNA repair mechanisms in FUS-associated ALS (Wang H. et al., 2018). Recently, TDP-43 was also shown to have a role in DNA repair (Mitra et al., 2019; Konopka et al., 2020), which is linked to oxidative stress (Guerrero et al., 2019). Collectively, these studies suggest that defects in DNA damage are a component of dysregulated redox homeostasis in ALS.

C9orf72 is a key regulator of lipid metabolism under conditions of cellular stress (Liu Y. et al., 2018). Loss of C9orf72 during starvation leads to dysregulated autophagy and increased *de novo* fatty acid synthesis (Liu Y. et al., 2018). Increased levels of free fatty acids and liquid droplets were detected in iPSC-derived motor neurons from C9orf72 ALS/FTD patients, suggesting the presence of dysregulated lipid metabolism by free fatty acid synthesis (Liu Y. et al., 2018). Similarly, increased levels of Lysosomal-associated membrane protein 1 (LAMP1) and NOX2 were detected in C9orf72 ALS/FTD patient iPSC-derived motor neurons, and in spinal cords of C9orf72 ALS patients (Liu Y. et al., 2018). Also, NOX2 was upregulated in embryonic fibroblasts obtained from C9orf72 knockout mice, suggesting that cellular redox conditions are dysregulated by depletion of C9orf72 (Liu Y. et al., 2018). From these studies, it is therefore tempting to speculate that C9orf72 is involved in the regulation of redox homeostasis through NOX2.

Proteomic analysis of the frontal cortex (area 8) in C9orf72 FTD patients revealed abnormal expression of proteins linked to the synthesis of ROS, suggesting the presence of redox dysregulation in these patients (Andrés-Benito et al., 2019). Also, modifiers of poly GR_100_ toxicity identified from an unbiased genome-wide nonessential yeast gene knockout study revealed dysregulation of mitochondrial and NADPH related metabolic pathways (Chai and Gitler, 2018). Consistent with these findings, another study demonstrated that increased oxygen and ATP consumption increased ROS, and induced mitochondria hyperpolarization in C9orf72 ALS patient-derived fibroblasts (Onesto et al., 2016). Overall, whilst these findings imply that increased production of ROS is associated with C9orf72-ALS, it is unclear whether loss of C9orf72 or expression of hexanucleotide repeat expansions disturbs redox homeostasis.

### SOD1

SOD1 is an important 32 kDa cytosolic antioxidant enzyme that facilitates the conversion of a superoxide anion radical ($O_{\text{2}}^{-}$) to H_2_O_2_ and oxygen (O_2_);

$$\text{2}O_{\text{2}}^{-}+\text{2}H+\to H_{\text{2}}O_{\text{2}}+O_{\text{2}}$$

(Mccord and Fridovich, 1969). Furthermore, SOD1 undergoes the cyclic reduction and oxidation of copper ions (Mccord and Fridovich, 1969), and it also inhibits oxidative inactivation of nitric oxide to prevent peroxynitrite formation (Harraz et al., 2008). More than 180 ALS-associated mutations in *SOD1* have been identified, and almost all these mutations are autosomal dominant. SOD1 was the first gene linked to ALS (Rosen et al., 1993), thus there has been intensive research into pathogenic mechanisms associated with mutant SOD1 (Rosen et al., 1993).

SOD1 normally regulates the NADPH oxidase-dependent production of $O_{\text{2}}^{-}$ by inhibiting the Rac1 signaling pathway. Rac1 is a member of the Rac family of guanosine triphosphate (GTP) phosphohydrolases (GTPases), which bind both guanosine diphosphate (GDP) and GTP, leading to its inactivation or activation, respectively. Rac1 normally cycles between the GTP and GDP bound states, depending on the redox state of Rac1. The interaction between SOD1 and Rac1 serves as a redox sensor for the regulation of NADPH oxidase (Harraz et al., 2008), and itself is redox-sensitive. Under oxidizing conditions, the SOD1-Rac1 GTP interaction is inhibited. However, during reducing conditions, SOD1 efficiently binds to Rac1 and activates the NOX2 signaling pathway (Harraz et al., 2008). However, the normal uncoupling of SOD1 from Rac1 is defective in SOD1^G93A^ mice, leading to Rac1 activation and hence inactivation of NADPH oxidase (Harraz et al., 2008).

The antioxidant properties of SOD1 and its relationship to neurodegeneration have been extensively studied in ALS (Proescher et al., 2008; Karch et al., 2009). Whilst loss of the normal SOD1 antioxidant enzymatic activity was initially proposed as a cause of neurodegeneration, further research implicated gain of a toxic function instead (Hu et al., 2003). Several studies have shown that misfolded, mutant SOD1 disrupts redox homeostasis in ALS by the abnormal production of ROS and RNS, inducing oxidative stress (Poon et al., 2005; Harraz et al., 2008). This is implicated in perturbing many cellular processes in both motor neurons and non-neuronal cells, including neuroinflammation (Bruijn et al., 2004; Wu et al., 2006; Marden et al., 2007). Also, both mutant and WT SOD1 produce cytotoxic levels of H_2_O_2_ *via* a cysteine redox regulation system (Bakavayev et al., 2019). Also, SOD1^G93A^ mice show significantly increased protein carbonyl levels in the spinal cord, including elevated carbonylation of mutant SOD1, which has been linked to motor neuron degeneration (Poon et al., 2005).

The gain-of-function of mutant SOD1 toxicity has been also related to its ability to generate oxidants, as well as to a higher sensitivity of the enzyme to oxidants. Oxidation of the solvent-exposed W32 residue in SOD1 in particular has been associated with aggregation of mutant SOD1 *in vitro* and *in vivo* (Taylor et al., 2007; Coelho et al., 2014; Duval et al., 2019). Furthermore, W32 can be oxidized by the carbonate radical produced by SOD1 bicarbonate-dependent peroxidase activity, leading to the formation of a SOD1 covalent dimer cross-linked by a di-tryptophan bond. The di-tryptophan cross-link may weaken the non-covalent bonds between the SOD1 monomers, triggering enzyme unfolding, oligomerization, and aggregation (Coelho et al., 2014). Finally, recent studies have suggested that the oxidation of lipids (such as polyunsaturated fatty acids and cholesterol) generates electrophilic compounds that modify Lys residues in SOD1, inducing its aggregation (Dantas et al., 2020).

ALS-associated SOD1 mutants produce free radicals (ONOO^−^ or OH^−^) and some mutants lose its catalytic activity, which in turn produces highly unstable intermediate products and tyrosine (Abe et al., 1997; Raoul et al., 2006). Nitro-tyrosine and nitrated proteins have also been detected in the CSF of both sALS and fALS patients, indicating the presence of redox imbalance in these tissues. Reversible phosphorylation of the SOD1 residue Ser39 in yeast and Thr40 in human cell lines (HEK293, Hep3B, A549, and MCF7 cell) by mTOR signaling moderates ROS levels, prevents oxidative damage, and regulates redox-dependent growth and survival (Tsang and Zheng, 2018; Tsang et al., 2018). Furthermore, a recent study comparing global to muscle-specific knockout of SOD1 in mice demonstrated differentially altered neuromuscular integrity and dysregulated redox pathways in both the nerve and muscle of these animals (Sakellariou et al., 2018). This study supports the notion of impaired redox signaling, rather than oxidative damage, in peripheral nerves playing a key role in muscle loss and muscle sarcopenia during aging (Sakellariou et al., 2018).

### NADPH Oxidase

Dysregulation of multiple transmembrane NOX proteins are implicated in ALS (Bedard and Krause, 2007; Marrali et al., 2014). Seven members of the NOX family of enzymes are known to exist in humans; NOX1, NOX2/gp91phox, NOX3, NOX4, NOX5, Duox1, and Duox2 (Bedard and Krause, 2007), and each has a specific tissue distribution and mechanism of activation (Leto et al., 2009). Importantly, activation of NOX generates the substrate $O_{\text{2}}^{-}$ for the reaction catalyzed by SOD1 (Mccord and Fridovich, 1969). NOX also controls the production of pro-inflammatory cytokines interleukin-1-β (IL-1β), and tumor necrosis factor-α (TNFα), which are elevated in the plasma and CSF of ALS patients (Poloni et al., 2000; Dengler et al., 2005) and in spinal motor neurons of SOD1^G93A^ (Hensley et al., 2003) and SOD1^G37R^ (Nguyen et al., 2001) mice. This correlates with enhanced activation of nuclear factor κ-light chain enhancer of activated B cells (NFκB; Nguyen et al., 2001), suggesting that NOX regulates neuroinflammation in ALS. NOX1 and NOX2 were also linked to dysregulation of redox homeostasis in SOD1^G93A^ mice (Marden et al., 2007). Similarly, NOX2 was also upregulated in SOD1^G93A^ mice and sALS patients (Kato et al., 2005). Importantly, deletion of *NOX2* and *NOX1* significantly delays disease progression and prolongs survival in SOD1^G93A^ mice (Bruijn et al., 2004; Poon et al., 2005; Marden et al., 2007). Furthermore, NOX2 activity was downregulated in peripheral neutrophils of ALS patients, which also correlated with improved survival and disease outcomes (Marrali et al., 2014). Also, elevated levels of NOX2, $O_{\text{2}}^{-}$ and inactivation of NOX1 in SOD1^G93A^ mice, induced by neuroinflammation, prolonged survival, and led to reduced ROS levels and oxidative stress in spinal cords of these animals (Wu et al., 2006). However, contradictory findings were obtained in another recent study, where genetic deletion of *NOX1* or *NOX2* did not improve survival in SOD1^G93A^ mice (Seredenina et al., 2016). Hence, whether deletion of NOX improves survival of SOD1^G93A^ mice remains unclear.

Whole-genome analysis of sALS patients identified *NOX4* as a potential genetic risk factor in sALS (Dunckley et al., 2007). This finding is intriguing because NOX4 also regulates ROS production, and lowering the levels of NOX4 decreases ROS production (Hordijk, 2006; Bedard and Krause, 2007). NOX4 is highly expressed in neurons but it is activated by different mechanisms compared to NOX1 and NOX2 (Hordijk, 2006). However, similar to NOX1 and NOX2, NOX4 can also be regulated by Rac1 and Akt, and protein kinase B signaling pathways (Gorin et al., 2003). Altered NOX-dependent redox stress induced by mutant SOD1 is also thought to be a secondary event associated with neuroinflammation and microgliosis (Boillée et al., 2006). The interplay between NOX, mutant SOD1, and microglia in the production of superoxide is therefore potentially important, and more studies in this area are warranted (Valdmanis et al., 2008; Zhou et al., 2020). However, conflicting evidence exists regarding the use of NOX as a therapeutic target in ALS (Marrali et al., 2014; Seredenina et al., 2016). More studies using specific NOX inhibitors or small molecules are therefore required to examine this possibility in more detail.

### Apurinic/Apyrimidinic Endonuclease

Apurinic/apyrimidinic endonuclease (APE1) is also known as redox effector factor 1 (RF1), human AP endonuclease 1 (HAP1), or apurinic/apyrimidinic endodeoxyribonuclease 1 (APEX1; Liu et al., 2005). APE1 is a 37 kDa ubiquitous multifunctional protein involved in the regulation of multiple transcription factors, including NF-κB, STAT3, AP-1, hypoxia-inducible factor-1 (HIF-1), and tumor protein 53 (p53; Chiueh, 2010). It has two major functions: DNA repair and redox regulation (Angkeow et al., 2002), and it is particularly important in neurons because they are highly susceptible to oxidative DNA damage (Coppedè, 2011). APE1 is particularly important in BER, a specific mechanism of DNA repair that eliminates damaged bases (oxidized or alkylated) generated by ROS (Hayward et al., 1999). APE1 is therefore considered to be neuroprotective because of its dual role in oxidative DNA damage and redox regulation (Coppedè, 2011).

APE1 is upregulated in motor neurons of ALS patients compared to age-matched controls (Shaikh and Martin, 2002). In addition, APE1 was also upregulated in astrocytes and spinal cord white matter in ALS patients (Shaikh and Martin, 2002). Furthermore, activity of the BER pathway was significantly increased in sALS patients (Kisby et al., 1997; Coppedè, 2011). In contrast, however, another study concluded that the activity of APE1 was reduced in sALS patients (Kisby et al., 1997). Furthermore, loss of immunoreactivity to APE1 was observed in spinal cords of pre-symptomatic SOD1^G93A^ mice, suggesting that reduced levels of APE1 in motor neurons precede neurodegeneration (Manabe et al., 2001; Nagano et al., 2002). The evidence for APE1 in ALS is therefore somewhat conflicting. Genomic DNA analysis identified several variants of APE1 (L104R, E126D, R237A, D283A, D148E, G306A, and G241R) that were over-represented in ALS patients compared to controls. However, these variants did not affect the DNA repair activity of APE1, and they do not contribute to the risk of developing sALS (Hayward et al., 1999; Hadi et al., 2000; Tomkins et al., 2000; Coppedè et al., 2010). APE1 also negatively regulates nuclear factor erythroid-related factor 2 (NRF2), a prominent antioxidant that is protective against oxidative damage triggered by injury and inflammation (Fishel et al., 2015).

APE1 is also associated with DNA damage induced by C9orf72 mutations. APE1 co-precipitates more with nucleophosmin (NPM1) in C9orf72 patient tissue lysates compared to controls (Farg et al., 2017), and overexpression of NPM1 inhibits apoptosis in neuronal cells expressing poly GR_100_ and poly PR_100_ (Farg et al., 2017). Also, genetic modifiers of poly GR toxicity in *Drosophila* identified APE1 and other DNA repair proteins (Ku80 and ERCC1) as suppressors of GR toxicity (Lopez-Gonzalez et al., 2019). A recent study also demonstrated mislocalization of APE1 from the nucleus to the cytoplasm as a possible trigger of oxidative DNA damage in spinal motor neurons expressing mutant SOD1^G93A^, despite upregulation of multiple DNA repair enzymes, suggesting that restricted localization of APE1 could inhibit redox homeostasis (Li J. et al., 2019).

APE1 interacts with several proteins involved in both DNA repair and redox regulation. Furthermore, the motor cortex of ALS patients contains epigenetic hypomethylation of the APE1 promoter (Kim et al., 2020). Apurinic/Apyrimidinic (AP) sites in the brain, spinal cord, and brainstem of ALS patients are vulnerable to DNA lesions induced by free radicals and intermediates (Kim et al., 2020). ROS can produce 50,000–200,000 AP sites in the genome of a mammalian cell every day, and it is estimated to produce significantly more AP sites in the brain (Atamna et al., 2000). Therefore, small molecules that mimic APE1 or elevate APE1 expression may be protective against DNA damage and dysregulated redox homeostasis in ALS. However, whilst this possibility has not been examined in detail, overexpression of human APE1 in brain and spinal cord motor neurons was protective against apoptosis and axotomy, in two independent mouse models of injury-induced neurodegeneration (Martin and Wong, 2017). The repair function of APE1 is protective by switching on antioxidant and cell survival mechanisms after oxidative DNA damage to neurons (Jiang et al., 2009).

### Peroxiredoxins

Prxs are a ubiquitous family of antioxidant enzymes that regulate peroxide and peroxynitrite levels in mammalian cells (Sanchez-Font et al., 2003). They are arguably the most important family of enzymes involved in peroxide metabolism because they are capable of reducing the levels of cellular H_2_O_2_ by 90% (Rhee, 2016). The family members in humans are classified, based on sequence homology and structural data, into six subfamilies, namely, Prx1, Prx2, Prx3, Prx4, Prx5, and Prx6.

There are now several lines of evidence that Prxs are dysregulated in ALS. Prx3, which is found in mitochondria, is downregulated in cells expressing SOD1^G37R^ and SOD1^G93A^ mutants, and in SOD1^G93A^ transgenic mice (Wood-Allum et al., 2006). Quantitative real-time PCR (Q-PCR) analyses also revealed downregulation of Prx3 in spinal cords of sALS and mutant SOD1 ALS patients, suggesting loss of redox regulation and these antioxidant defense mechanisms in ALS (Wood-Allum et al., 2006). However, Prx6 was upregulated in spinal motor neurons of SOD1^G93A^ mice (Strey et al., 2004) and Prx 1 was upregulated in NSC-34 cells expressing SOD1^G93A^ or SOD1^G37R^ mutants (Allen et al., 2003). Similarly, Prx2, Prx3, catalase, and Prx6 were upregulated in SOD1^G93A^ mice compared to WT controls (Pharaoh et al., 2019). In contrast, Prx3 and Prx4 were downregulated in the presence of mutant SOD1^G93A^ in NSC-34 cells (Kirby et al., 2005), and immunohistochemical analysis of motor neurons from sALS and fALS patients revealed negative immunoreactivity for Prx2 and glutathione peroxidase-l (Kato et al., 2005). These findings together imply that upregulation of Prxs renders motor neurons less susceptible to neurodegeneration, whereas breakdown of this redox system at late disease stages induces neuronal degeneration and accelerates disease progression (Kato et al., 2005). Prx3 and Prx5 were also present in hSOD1^G93A^ aggregates in cells, suggesting that Prxs may also be involved in protein folding (Wood-Allum et al., 2006; Knoops et al., 2016). Further evidence for this notion comes from observations that Prx5 downregulation correlates with downregulation of molecular chaperones, and upregulation of proteins associated with neuroinflammation (Knoops et al., 2016). Therefore, these studies reveal that several peroxiredoxins are dysregulated in ALS models, but how this contributes to pathology is unknown.

### TMX2

TMX2, which is localized in the MAM compartment, acts as an important sensor of redox conditions and is crucial for the maintenance of cellular redox homeostasis (Vandervore et al., 2019). TMX2 functions in neuronal differentiation, dendritic, and axonal growth, and its dysregulation is linked to severe developmental abnormalities in the brain (Vandervore et al., 2019). CRISPR-Cas9 whole genome-wide gene-knockout screens for suppressors and enhancers of C9orf72 DPRs in human cells revealed TMX2 as an important protective modifier against toxicity (Kramer et al., 2018). This study suggested that depletion of TMX2 using single guide (sg) RNA in K562 cells and mouse primary cortical neurons was sufficient to suppress PR_20_ induced cellular toxicity (Kramer et al., 2018). Furthermore, transcriptomics performed from TMX2 deleted PR_20_ and GR_20_ expressing neurons revealed upregulation of pro-survival unfolded protein response (UPR) pathway genes and downregulation of calcium-binding and apoptotic genes. These findings, therefore, suggest that loss of TMX2 is protective against DPR toxicity by modulating ER stress (Kramer et al., 2018).

### Activator Protein 1

AP-1 is a redox-sensitive transcription factor that regulates gene expression in response to various stimuli, including cytokines, growth factors, and cellular stress. It is controlled by the MAP kinase cascade (Gomez Del Arco et al., 1997) and is therefore involved in a range of cellular processes, including cell differentiation, growth, and proliferation. Activation of AP-1 by mutant SOD1 in NSC-34 cells mediates upregulation of Bcl2-A1, which regulates apoptosis (Kale et al., 2018). This finding, therefore, suggests that AP-1 drives the regulation of apoptosis in motor neurons (Iaccarino et al., 2011). AP1 is a complex of several proteins including JUN, which is upregulated in motor neurons derived from SOD1 patients compared to other neurons, providing potential mechanistic insights into the selective degeneration of motor neurons in ALS (Bhinge et al., 2017). Furthermore, another component of the API complex, FOSL1, is highly expressed in IPSC-derived motor neurons from SOD1 patients carrying the E100G mutation (Bhinge et al., 2017) compared to isogenic controls, suggesting that the AP1 complex is a driver for neurodegeneration. Also, the AP1 complex FBJ osteosarcoma oncogene (c-FOS) was upregulated in neuronal cells expressing SOD1 mutations, suggesting dysregulation of these antioxidant response proteins (Kirby et al., 2005). However, a direct relation between AP-1 and redox regulation in ALS has not been defined.

### PDI Family of Proteins

The ER is a redox-regulated organelle that maintains redox homeostasis and facilitates protein folding (Sevier and Kaiser, 2008). However, protein misfolding within the ER triggers ER stress, which induces the UPR, a distinct signaling pathway that aims to relieve this stress (Matus et al., 2013; Hetz and Saxena, 2017). While initially protective, prolonged UPR induces apoptosis. PDIA1 (also known as PDI) is the prototype of the PDI family of ER chaperones which are induced during the UPR. The redox regulation of PDI is a crucial component of the maintenance of a balanced redox environment, and inhibition of its enzymatic activity will lead to important consequences for the cell. PDI has now been implicated in several neurodegenerative disorders, including ALS (Perri et al., 2016). Recent studies showing the protective effect of the redox activity of PDI in cellular and zebrafish models of ALS have placed redox dysregulation centrally in ALS, implying that it has a much broader role than previously realized.

As well as general chaperone activity, PDI family members possess oxidoreductase activity, which mediates the formation of protein disulfide bonds by cysteine residues located within its active site. Hence PDI proteins play a critical role in regulating the intracellular redox state, redox signaling, and in preventing protein misfolding and/or aggregation (Wang et al., 2015). The cysteine residues of PDI family members such as PDI, ERp57, and ERp72 contain redox-sensitive side chains and may become oxidized during redox dysregulation (Valle and Carrì, 2017). These residues are also actively modified by post-translational regulation, including disulfide formation and S-nitrosylation (Paulsen and Carroll, 2013; Fra et al., 2017). Both the chaperone activity and the overall conformation of human PDI are redox-regulated. Conformational changes in PDI alter its compact conformation and expose the normally shielded hydrophobic regions, which regulates its chaperone activity (Wang et al., 2012, 2015). GSH also regulates PDI functions and facilitates the redox activity of PDI during protein folding (Chakravarthi et al., 2006).

There is now growing evidence for a role of PDI in ALS (Parakh and Atkin, 2015; Parakh et al., 2020). PDI levels are upregulated in transgenic models of ALS and spinal cord tissues of ALS patients (Walker et al., 2010; Honjo et al., 2011; Jeon et al., 2014). Novel roles for PDI proteins were also recently identified in neurons, in mediating motor function and neuronal connectivity (Castillo et al., 2015; Woehlbier et al., 2016). Mutations in PDI and ERp57 were also described in ALS patients, but are thought to be more of a risk factor than directly causative of neurodegeneration (Woehlbier et al., 2016). Recently, the redox function of PDI, in contrast to its chaperone function, was shown to be protective against multiple cellular processes that dysfunction in ALS; protein misfolding, mislocalization of TDP-43 to the cytoplasm, ER stress, inhibition of ER-Golgi transport, and apoptosis, in neuronal cells expressing pathological forms of TDP-43 or SOD1 (Parakh et al., 2020). Furthermore, the redox activity of PDI, but not its chaperone function, rescued motor dysfunction and axonopathy in zebrafish models of ALS expressing mutant SOD1, together implying that PDI has an important role both *in vitro* and *in vivo* (Parakh et al., 2020). In contrast, the PDI ALS-mutants (D292N and R300H) lack this redox activity and were not protective against ALS phenotypes, further confirming the importance of the redox activity of PDI in ALS (Parakh et al., 2020). These findings, therefore, implicate redox homeostasis as a central and dominant feature of ALS and suggest that regulation of the neuronal redox environment has a much broader link to neurodegeneration than previously recognized.

The upregulation of PDI in ALS suggests that a cellular defensive mechanism is triggered against redox dysfunction. However, there is evidence that the normal protective function of PDI is inhibited in neurodegeneration (Walker et al., 2010). Modifications of active site thiol groups by two redox-dependent aberrant post-translational modifications, S-glutathionylation and S-nitrosylation, lead to inactivation of the normal enzymatic activity of PDI (Walker et al., 2010). Both of these direct oxidation processes affect crucial active site cysteine residues and result in a loss of enzymatic activity (Halloran et al., 2013). S-nitrosylation involves the transfer of NO to one or more cysteine thiol groups and it occurs when there is an increased production of RNS during oxidative stress (Conway and Harris, 2015). This process represents a prominent redox reaction mediating NO signaling under both physiological and pathophysiological conditions (Halloran et al., 2013). PDI is S-nitrosylated in lumbar spinal cords of sporadic ALS patients (Walker et al., 2010), which abrogates PDI-mediated attenuation of neuronal cell death triggered by ER stress or misfolded proteins (Uehara et al., 2006). Furthermore, in the presence of S-nitrosylated PDI, the formation of mutant SOD1 aggregates increases *in vitro* (Jeon et al., 2014). These findings suggest that loss of PDI functional activity can directly lead to apoptosis, or indirectly, to a range of cellular abnormalities, such as oxidative stress, protein misfolding, as well as cell death (Jeon et al., 2014). Hence, these data imply that loss of PDI function contributes to pathophysiology in ALS and that PDI controls the cellular redox environment in the development of neurodegeneration.

## Indirect Evidence for A Role of Redox Dysregulation in Als

**Figure 2:**
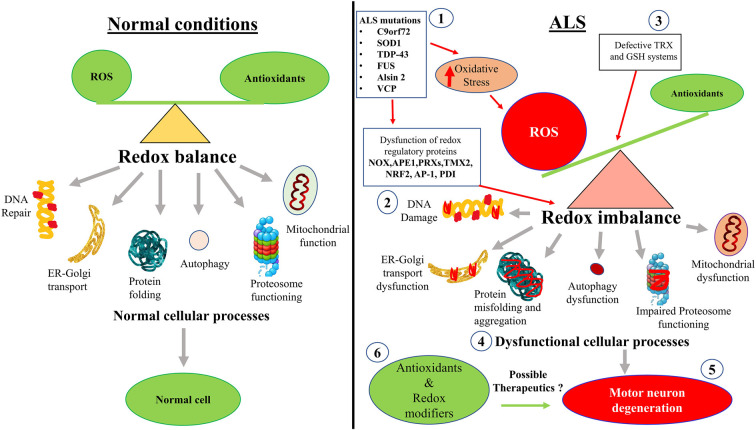
Hypothetical schematic diagram illustrating how redox imbalance may induce neurodegeneration in ALS. (1) ALS mutant proteins induce oxidative stress and damage, which (2) dysregulates redox regulatory proteins and (3) impairs the TRX and GSH systems. This leads to increased ROS production and redox imbalance in neurons. (4) Redox imbalance subsequently induces dysfunction to mitochondria, the proteasome and autophagy, cellular trafficking, protein misfolding and aggregation, and inhibits DNA repair. (5) Dysregulation of these cellular processes induces motor neuron degeneration in ALS. (6) Redox modifiers and antioxidants have been trialled clinically. However, interventions that control redox regulatory processes may be more beneficial than administering the antioxidants themselves. Diagram not to scale.

As well as the studies described above, there is also evidence that cellular processes associated with redox homeostasis are dysregulated in ALS (Figure 2). These more indirect mechanisms associated with redox perturbations in ALS are described in detail below.

## Cellular Processes Linked to Redox Imbalance That Are Dysregulated in Als

### Protein Misfolding

The presence of misfolded proteins is known to induce oxidative stress. However, there is also evidence that oxidative stress induces protein misfolding. Mutant SOD1 forms inclusions in the presence of oxidative stress and WT SOD1 misfolds when the redox environment is dysregulated (Oeda et al., 2001). BSO inhibits glutathione synthesis (Hamilos and Wedner, 1985; Spitz et al., 1995) and treatment of neuronal cells expressing mutant TDP-43 with BSO leads to increased inclusion formation (Parakh et al., 2020). Importantly, WT forms of both SOD1 and TDP-43 form inclusions following BSO treatment. Similarly, SOD WT misfolds and develops a similar conformation to mutant SOD1, leading to aggregation and the gain of toxic functions *in vitro* (Bosco et al., 2010; Guareschi et al., 2012; Parakh et al., 2020). These studies, therefore, highlight redox dysregulation as an important trigger for protein misfolding, which is central to neurodegeneration in ALS.

Redox dysregulation is also linked to protein misfolding by the production of aberrant, non-native disulfide bonds in both mutant SOD1 and TDP-43, which leads to the formation of inclusions and induces toxicity. In mutant SOD1, these aberrant disulfide bonds involve cysteine residues Cys 6 and Cys111, and in both mutant and WT TDP-43, cysteines Cys173, 175, 198, and 244 in the RNA-recognition motif (RRM) are involved (Cohen et al., 2012; Shodai et al., 2013). Compared with WT SOD1, disease-linked mutant SOD1 proteins readily form monomers by reduction of the disulfide bond between Cys-57 and Cys-146 or by demetallation at the dimer interface (Tiwari and Hayward, 2003; Rakhit et al., 2004). Due to this monomerization, mutant SOD1 has an increased propensity to misfold (Rakhit et al., 2004; Kerman et al., 2010). Several previous studies have concluded that aberrant, non-native disulfide bonds involving Cys-6 and Cys-111 result in inclusion formation and disulfide reduction (Deng et al., 2006; Furukawa et al., 2006; Wang et al., 2006; Niwa et al., 2007) and the induction of both ER stress and toxicity (Alvarez-Zaldiernas et al., 2016; Xu G. et al., 2016; Perri et al., 2020). Moreover, these aberrant disulfide bonds have also been identified *in vivo* (Karch et al., 2009; Medinas et al., 2018). Similarly, aberrant disulfide cross-linking leads to misfolding and subcellular mislocalization of TDP-43 (Barmada et al., 2010; Cohen et al., 2012), which is induced by dysregulation of redox conditions. Hence, oxidative stress promotes the formation of these non-native disulfide bonds which leads to aggregation of both mutant SOD1 and TDP-43 (Cohen et al., 2012; Fukai and Ushio-Fukai, 2011).

Another link between protein misfolding and redox mechanisms is illustrated by the presence of important redox proteins in the misfolded protein inclusions in ALS. PDI associates with misfolded protein inclusions in patients with ALS (Honjo et al., 2011; Parakh et al., 2018a) cellular models (Farg et al., 2012; Jeon et al., 2014), and canine degenerative myelopathy (DM; Chang et al., 2019), and both PDI and ERp57 inhibit the formation of mutant SOD1 inclusions in neuronal cells (Walker et al., 2010; Parakh et al., 2018a). Furthermore, Keap1, a cysteine rich protein which binds to NRF2 and regulates oxidative and electrophilic stress, was co-localized with intracellular misfolded protein inclusions in motor neurons in the spinal cord of ALS patients. Moreover, in the motor cortex of ALS patients, the levels of NRF2 mRNA and protein were reduced, whereas Keap1 mRNA expression was increased compared to control patients (Sarlette et al., 2008; Tanji et al., 2013), suggesting that the NRF2-EpRE pathway is dysfunctional in ALS.

### Mitochondrial Damage

Mitochondria are the major cellular site of ROS production and damage to mitochondrial structure or function increases oxidative stress (Albers and Beal, 2000; Carrí et al., 2003). Mitochondrial impairment and dysregulation of mitochondrial proteins are present in postmortem brain and spinal cord tissues of ALS patients and SOD1^G93A^ mice (Carrí et al., 2003; Ferri et al., 2006; Tan et al., 2014). Similarly, WT TDP-43 interacts with several mitochondrial proteins that are crucial for mitophagy and mitochondrial dynamics (Davis et al., 2018). Expression of ALS-associated mutant TDP-43^A315T^ also leads to mitochondrial abnormalities in cell culture (Gao et al., 2019; Wang P. et al., 2019). Furthermore, mitochondrial damage is present early in disease course in TDP-43^A315T^ mice and increases motor neuron vulnerability (Gautam et al., 2019), consistent with dysregulation of the redox system early in neurodegeneration. ALS-associated mutant TDP-43 is known to be aberrantly localized in mitochondria, but suppressing its mitochondrial localization protects against neurotoxicity in TDP-43^A315T^ mice (Wang et al., 2016). Similarly, motor and cognitive functions in TDP-43^A315T^ mice are improved by inhibiting the mitochondrial localization of TDP-43 (Wang et al., 2017). However, contradictory findings were obtained in a recent study which concluded that mutant TDP-43^A315T^ does not impair mitochondrial bioenergetics *in vitro* and *in vivo* (Kawamata et al., 2017).

The C9orf72 DPRs also interact with mitochondrial proteins, resulting in mitochondrial dysfunction, inflammation, and neurotoxicity (Gendron and Petrucelli, 2018). Elevated production of ROS in C9orf72-associated ALS is also associated with abnormalities in mitochondrial function and neuroinflammation (Briehl et al., 2014; Alvarez-Zaldiernas et al., 2016). C9orf72 ALS/FTD-associated poly (GR)_80_ DPRs interact with Atp5a1, which compromises mitochondrial functions in mice due to increased ROS production (Choi et al., 2019). Also, poly (GR)_80_ interacts with mitochondrial ribosomal proteins, inducing mitochondrial dysfunction, in motor neurons differentiated from C9orf72 patient iPSCs, compared to controls (Lopez-Gonzalez et al., 2016). Induction of oxidative stress also induced more DNA damage in iPSC-derived C9orf72 motor neurons than controls, in an age-dependent manner (Lopez-Gonzalez et al., 2016). Furthermore, in the same study, expression of poly (GR)_80_ in neurons increased ROS levels, inducing oxidative stress and dysregulating redox conditions in C9orf72 iPSCs (Lopez-Gonzalez et al., 2016). Pharmacological reduction of oxidative stress by administering Trolox, a water-soluble antioxidant and vitamin E analog, also partially rescued DNA damage and cellular toxicity in *Drosophila* expressing C9orf72 DPRs (Lopez-Gonzalez et al., 2016). Furthermore, myogenic progenitors derived from C9orf72 ALS patients displayed increased susceptibility to oxidative stress and dysregulation of mitochondrial genes, and these events were associated with mitochondrial abnormalities and toxicity (Lynch et al., 2019).

Mitochondria connect to the ER at multiple contact sites to form the MAMs. Mutations in *C9orf72, TDP-43, VAPB, VCP*, and *FUS* are known to disturb these ER-mitochondria associations and signaling (Stoica et al., 2014; Zhang et al., 2017; Lau et al., 2018). FUS is known to interact with HSP60, which is associated with mitochondrial abnormalities, and these defects have been identified in FUS-FTLD patients (Deng et al., 2015). Mutant FUS^R495X^ dysregulates the expression of genes associated with oxidative mitochondrial metabolism in neurons, and significantly reduces the size of mitochondria, which induces neurotoxicity (Nakaya and Maragkakis, 2018). Similarly, ER-mitochondria associations and VAPB-PTPIP51 interactions are disrupted by ALS-associated mutant FUS (Stoica et al., 2016), which results in increased production of ROS. The ATP synthase beta subunit, a mitochondrial enzyme involved in redox regulation, associates with FUS, inducing the mitochondrial UPR in cellular and transgenic *Drosophila* models of ALS (Deng et al., 2018). Similarly, abnormal accumulation and aggregation of mitochondria in the inter-myofibrillar space was detected in iPSCs derived from an ALS patient bearing a VCP mutation (Bartolome et al., 2013; Hall et al., 2017). Furthermore, heterozygous knock-in of mutant VCP^R155H^ in mice leads to alterations in mitochondrial respiratory complex activity (Yin et al., 2012). Fibroblasts obtained from ALS patients carrying the CHCHD10 mutation S59L display respiratory chain deficiency, ultrastructural alterations, and fragmentation of the mitochondrial network (Bannwarth et al., 2014; Mccann et al., 2020). Therefore, together these studies imply that mutant proteins linked to ALS induce damage to mitochondrial structure, impair its function, dysregulate energy metabolism, and disturb redox homeostasis.

### Neuroinflammation

Although motor neurons specifically degenerate in ALS, increasing evidence implies that non-neuronal cells, such as astrocytes (Lee et al., 2016), microglia (Henkel et al., 2009), and oligodendrocytes (Li J. et al., 2016), directly contribute to neurodegeneration by a non-cell-autonomous mechanism (Radford et al., 2015). This results in the appearance of reactive microglia and astroglia which is referred to as neuroinflammation (Radford et al., 2015). Mutant ALS proteins trigger microglial activation, increase the levels of ROS, and induce neurotoxicity (Henkel et al., 2009). Microglia exist in two states, resting and activated, and their activation represents a continuum between the two classical phenotypes; neuroprotective M2 vs. neurotoxic M1 (Liao et al., 2012; Chiu et al., 2013). Two different microglial phenotypes have also been described in SOD1^G93A^ transgenic mice. Mutant SOD1^G93A^ and SOD1^G85R^ activate microglia and increase the levels of ROS and other pro-inflammatory cytokines (Zhao et al., 2010). Transcriptome analysis of microglia isolated from SOD1^G93A^ mice revealed increases in activated pro-inflammatory *Igf1*, Progranulin, *Trem2*, cytokines, and neurotoxic factor MMP-12 (Chiu et al., 2013). Also, mutant SOD1^G93A^ and SOD1^L8Q^ stimulate Rac1-GTP activation of NOX and the production of ROS, unlike WT SOD1 (Boillée et al., 2006; Beers et al., 2011). Members of the NOX enzyme family catalyze the formation of ROS and are implicated as mediators of neurodegeneration induced by neuroinflammation in ALS (Calvo et al., 2014). Therefore, together these findings suggest the interplay between NOX, ROS, the redox state of Rac1 and SOD1 contributes to neuroinflammation and associated toxicity in ALS.

M1 microglial cells are also activated by inflammatory stimuli. Mutant TDP-43 activates M1 microglia and upregulates pro-inflammatory mediators NOX2, TNF-α, and IL-1β (Beers et al., 2011; Liao et al., 2012; Chiu et al., 2013). Consistent with these findings, glial cells express more endogenous TDP-43 after treatment with lipopolysaccharide (LPS) or ROS, and produce more pro-inflammatory cytokines and neurotoxic mediators (Swarup et al., 2011). Furthermore, expression of WT, ALS-associated mutant, or truncated forms of TDP-43, promote CD14-mediated activation of microglia through NF-κB signaling and NLRP3 inflammasomes (Zhao et al., 2015). WT FUS also activates NF-κB, the master regulator of inflammation, and induces expression of both pro-inflammatory markers and redox signaling proteins in microglia (Frakes et al., 2014; Geloso et al., 2017; Ajmone-Cat et al., 2019).

C9orf72 repeat expansions activate microglia and astrocytes, as well as initiate the formation of innate immune inflammasome complexes and activation of intracellular receptors responsible for inducing inflammation. Astrocytes derived from C9orf72 and sporadic ALS patients were found to be toxic *in vitro* and *in vivo* to motor neurons, by a non-cell-autonomous mechanism (Di Giorgio et al., 2007; Yamanaka et al., 2008; Haidet-Phillips et al., 2011). This toxicity involved either secretion of neurotoxic factors or loss of astrocytic support functions (Meyer et al., 2014; Madill et al., 2017). Post-mortem analyses of spinal cord tissue sections from sALS patients revealed increases in markers of lipid peroxidation and protein glycoxidation, resulting in oxidative damage in both neurons and non-neuronal cells (Shibata et al., 2001). However, a recent study revealed that iPSC-derived astrocytes obtained from C9orf72 ALS patients display increased oxidative stress and senescence (Birger et al., 2019). Furthermore, motor neurons cultured using conditioned media from iPSC-derived C9orf72 astrocytes exhibit increased oxidative stress (Birger et al., 2019). Also, mutant C9orf72-derived astrocytes downregulate secretion of several antioxidants and induce cellular senescence (Birger et al., 2019). Despite these findings, however, it remains unclear whether neurotoxicity is induced by astrocyte disturbances to redox homeostasis in C9orf72 ALS. Furthermore, whilst loss of C9orf72 in mice models disturbs microglial function, resulting in age-related neuroinflammation, this was not sufficient to cause neurodegeneration in C9orf72 knockout mice (Lall and Baloh, 2017). Collectively, these studies imply mutant ALS proteins produce inflammatory cytokines, disturb redox homeostasis, and increase neuroinflammation and toxicity. However, it remains unclear whether activation of inflammatory cytokines in C9orf72 ALS models is directly linked to redox regulation.

### Cellular Trafficking Defects

Haploinsufficiency is increasingly implicated as a disease mechanism in ALS, which is characterized by reduced expression of C9orf72 in ALS patients. C9orf72 is a member of the DENN domain family of proteins (Zhang et al., 2012) and it was initially found to interact with Rabs 1, 5, 7 and 11, in the regulation of endocytosis and autophagy in neuronal cells (Farg et al., 2014). However, several later studies have now shown that C9orf72 also interacts with many other Rabs; Rab 1a, 1b, Rab3 (a, b, c, d), Rab5a, Rab7a Rab7L1, 8a, 8b, 10, 13, 15, 18, 19, 27a, 38, 40a and 42 (Gitler and Tsuiji, 2016; Tang, 2016; Webster et al., 2016; Aoki et al., 2017; Gao et al., 2017). C9orf72 was also shown to be a Rab guanine exchange factor (GEF) involved in the modulation of Rab activity (Iyer et al., 2018), which regulates NOX2 recruitment to phagosomes in dendritic cells (Jancic et al., 2007). C9orf72 also forms a complex with SMCR8 and WDR41 in the regulation of autophagy, and it is also implicated in autoimmunity, immune dysregulation, endocytosis, and lysosome homeostasis (Atanasio et al., 2016; O’Rourke et al., 2016; Sullivan et al., 2016; Webster et al., 2016; Yang et al., 2016; Corrionero and Horvitz, 2018; Zhang et al., 2018). Lysosomal accumulation was observed in SMCR8 deficient macrophages from Smcr8^−/−mice^, possibly because of increased ROS (Mcalpine et al., 2018). Rab-mediated cellular trafficking defects are also known to be induced by mutant forms of SOD1, TDP-43, and FUS (Soo et al., 2015; Parakh et al., 2018b).

The *ALS2* gene is mutated in autosomal recessive forms of juvenile-onset ALS (Yang et al., 2001). *ALS2* encodes alsin, which functions as a guanine nucleotide exchange factor (GEF) for the small GTPase Rab5. There are two isoforms, long and short. The long isoform consists of three independent GEF-like domains (RCC1 domain, PH domain, and VPS9 domain) whereas the short contains the RCC1 domain (Yang et al., 2001). The longer isoform alone is known to act as a Rab GEF or Rab activating protein for Rab5 and Rac1GTPases, and it functions in endocytic mechanisms, endosomal dynamics, and micropinocytosis (Topp et al., 2004; Otomo et al., 2008). An important aspect of redox signaling is the localization of redox-active processes within distinct microenvironments of the cell, and redox-active endosomes (redoxosomes) are one key example of this. Redoxosomes contain redox proteins that are involved in transmitting ROS signals from interior to outer membranes, and they regulate ROS as a secondary messenger (Oakley et al., 2009).

Interestingly, alsin and SOD1 are both effectors of Rac1GTPases (Kanekura et al., 2004). The interplay between SOD1, alsin, and Rac1 in endosome formation and trafficking is particularly intriguing in the context of NOX dependent production of IL-1β and TNFα. Hence this is also relevant to SOD1-mediated redoxosomal signaling defects in ALS (Kanekura et al., 2005; Otomo et al., 2008; Li et al., 2011). Furthermore, alsin also interacts with mutant SOD1 and Rac1 to inhibit hyperactivation of NOX and reduce ROS production (Jacquier et al., 2006; Li et al., 2011). It is therefore tempting to speculate that defects in the interaction between SOD1, Rac1, and alsin 2 influence redox signaling at the endosomal level.

There is also evidence that alsin is protective against oxidative stress and may be involved in redox regulation. First, overexpression of the alsin long isoform protects against motor neuron toxicity induced by expression of A4T, or mutant SOD1^G85R^ or SOD1^G93A^ (Kanekura et al., 2004). Second, knockout of alsin in mice is not sufficient to trigger motor neuron degeneration, but neurons cultured from these animals are more susceptible to oxidative stress (Cai et al., 2005). Furthermore, these mice exhibit defects in endosomal trafficking (Devon et al., 2006; Hadano et al., 2010), indicating that alsin could be a component of the redox-sensing mechanisms that inhibit NOX signaling. Together these findings, therefore, suggest that alsin protects cells from oxidative stress and could be a redox sensing protein, similar to Rab5, Rac1, and SOD1.

## Modifiers of Redox Regulation as A Therapeutic Target for Als

Only two drugs are currently approved by the USA Food and Drug Administration (US FDA-FDA) for ALS treatment. Given the extensive evidence linking redox dysfunction to ALS, it is not surprising that several redox-active molecules have been trialed as potential therapeutic agents. However, whilst multiple compounds targeting redox regulation have been reported to slow disease progression in SOD1^G93A^ mice (Dash et al., 2018), they have subsequently failed to enhance survival or improve motor function in ALS patients in clinical trials. Efforts to modulate GSH directly have failed, due to limits of solubility, absorption, stability, and the short half-life of GSH. Moreover, direct administration of cysteine to increase GSH is not a viable option, because of its poor absorption and toxicity at high doses (Johnson et al., 2012). Nevertheless, the second FDA-approved drug for ALS, edaravone, is a strong antioxidant that inhibits oxidative stress and is a potent scavenger of free radicals, highlighting the importance of redox regulation in disease. The ALS Functional Rating Scale-Revised (ALSFRS-R) is a scale that determines the progression and severity of ALS patients and is widely used in clinical trials (Rooney et al., 2017). Unfortunately, edaravone can only be used in a small subset of early-stage ALS patients (grade 1 or 2 in the Japan ALS Severity Classification, scoring at least 2 points on all 12 items of ALSFRS-R; Dash et al., 2018). Furthermore, it is not yet available orally. Riluzole was the first FDA-approved compound for ALS (in 1995), which inhibits glutamatergic neurotransmission and thus inhibits excitotoxicity (Dharmadasa and Kiernan, 2018; Dash et al., 2018). However, administration of riluzole statistically only improves survival in ALS patients up to 60 days. Hence both compounds are not particularly effective (Dharmadasa and Kiernan, 2018; Dash et al., 2018). There is therefore a current need to develop much more successful therapeutics. The section below will discuss therapeutics strategies that have targeted modifiers of redox regulation in pre-clinical models of ALS and/or clinical trials. Table 3 summarizes the studies discussed below.

**Table 3:** List of potent redox modifiers trialled in ALS.

Drug	Properties	Mechanism of action	Trial/Study	Results	References
**(A) CLINICAL TRIALS**			
Alpha-tocopherol (Vitamin E) 500 mg	Antioxidant	Reduces oxidative stress	Randomized placebo-controlled clinical trial (RCT)	No significant improvement in disease progression or survival	Desnuelle et al. (2001) and Graf et al. (2005)
Higher doses of Vitamin E (5,000 mg)	Antioxidant	Reduces oxidative stress	Phase III RCT	No significant improvement in disease progression or survival	Graf et al. (2005)
Nanocurcumin or Vitamin E as add-on therapy with Riluzole	Anticancer agent and antioxidant	Vitamin E reduces oxidative stress and Riluzole increases glutamate uptake	Pilot RCT	No significant improvement in motor function of ALS patients	Ahmadi et al. (2018)
Vitamin C and Carotenoids Supplementation	Antioxidant	Reduces oxidative stress	Pooled results from 5 different cohort studies	Does not reduce the risk of developing ALS	Fitzgerald et al. (2013)
Coenzyme Q10 (CoQ10)	Antioxidant and co-factor in the ETC	Reduces oxidative stress and mitochondrial impairment	Open-label dose-escalation trial	No significant improvement in ALSFRS-R score	Ferrante et al. (2005)
Dexpramipexole	Antioxidant and apoptosis inhibitor	Reduces oxidative stress	Phase III RCT	No significant improvement in motor function of ALS patients	Cudkowicz et al. (2013)
Combination of Vitamin C, E, selegiline, selenium, and L-methionine	Combination of antioxidants	Reduces oxidative stress	All randomized or quasi-randomized controlled trials	No significant improvement in disease progression or survival in ALS patients	Orrell et al. (2008)
Edaravone	Strong antioxidant	Reduces oxidative stress	Phase 1, II, III RCTs	Improves motor function by 33% compared to control patients. Significant improvement in survival in a subset of ALS patients	Writing Group on Behalf of the Edaravone (MCI-186) ALS 19 Study Group (2017a), Writing Group; Edaravone (MCI-186) ALS 19 Study Group (2017b), Abe et al. (2014), and Takei et al. (2017a)
Curcumin (600 mg/day, Brainoil)	Antioxidant, anti-inflammatory, anti-cancer agent	Reduces oxidative stress and neuroinflammation	Double-blind controlled trial	Reduces oxidative stress and improves aerobic metabolism	Chico et al. (2018)
EH301, a combination of PT and NR	Combination of antioxidant and anti-aging agent	Improves mitochondrial oxidative metabolism	Pilot RCT	Slows disease progression in a small number of ALS patients	De La Rubia et al. (2019)
**(B) PRE-CLINICAL MODELS**			
H_2_S	Antioxidant	Increases Ca^2+^ levels, improves mitochondrial functions, and inhibits SOD1 aggregation	SOD1^G93A^ mice	Effective against mitochondrial dysfunction	Pratt et al. (2012) and Paul and Snyder (2018)
Fisetin and 7,8-Dihydroxyflavone	Antioxidant	Reduces ROS production and activates the ERK signaling pathway	SOD1^G93A^ mice	Reduces ROS production and neurodegeneration	Korkmaz et al. (2014)
Resveratrol	Antioxidant, Anti-aging agent	Inhibits oxidative stress	SOD1^G93A^ mice	Significant improvement in motor function and survival	Mancuso et al. (2014) and Song et al. (2014)
Epigallocatechin	Antioxidant	Inhibits oxidative stress and ROS production	SOD1^G93A^ mice	Significant improvement in motor function and survival	Koh et al. (2004), 2006) and Xu et al. (2006)
CPN-9	NRF2 activator	Inhibits ROS production	SOD1^H46R^ mice	Improves motor function and delays disease progression	Kanno et al. (2012)
NR, a form of Vitamin B_3_	Antioxidant	Inhibits oxidative stress, activates mitochondrial UPR and SIRT6 expression, reduces neuroinflammation	SOD1^G93A^ mice	Delays motor neuron degeneration, reduces neuroinflammation in the spinal cord, and slightly prolongs survival	Zhou et al. (2020)
Arimoclomol	Inducer of heat shock proteins, indirect antioxidant	Reduces mutant SOD1 aggregation and enhances protein folding	SOD1^G93A^ mice	Protective against toxic mutant SOD1 aggregates, delays disease progression, and improves motor functions	Kieran et al. (2004), Kalmar et al. (2008), and Lanka et al. (2009)
**(C) CELL CULTURE AND DROSOPHILA MODELS OF ALS**			
γ-Oryzanol	Antioxidant, Anti-inflammatory	Reduces oxidative stress and neuroinflammation	SOD1^G85R^ *Drosophila* and cell culture models	Reduces oxidative stress ad neurotoxicity	Zhang et al. (2019a)
Urate	Antioxidant	Reduces astrocyte induced toxicity and oxidative stress	Mutant SOD1^G93A^ cell culture and Drosophila model of ALS	Neuroprotection against motor neuron toxicity induced by mutant SOD1^G93A^	Bakshi et al. (2018) and Zhang et al. (2019b)
Lipoic acid	Antioxidant, Anti-inflammatory	Inhibit oxidative stress and reduces neuroinflammation associated toxicity	SOD1^G93A^ and SOD1^G85R^ *Drosophila* and cell culture models	Attenuates oxidative stress and protects against neurotoxicity	Moura et al. (2015), Saleh et al. (2017), and Wang T. et al. (2018)
Diallyl trisulfide	Antioxidant	Increases the expression of HO-1 and NQO1 to prevent oxidative stress	TDP-43 ^Q331K^ and TDP-43 ^M337V^ cell culture models of ALS	Protects from motor neuron toxicity	Liu C. et al. (2018)

## Clinical Trials

Vitamin E is known to regulate redox balance, and a randomized placebo-controlled clinical trial examined the effect of administering 500 mg alpha-tocopherol (the primary form of vitamin E used by humans) to ALS patients, along with riluzole. However, no beneficial effects on disease progression or survival were observed in these patients, despite changes in biochemical markers of oxidative stress (Desnuelle et al., 2001; Galbussera et al., 2006). Similarly, another phase III clinical trial using higher doses of vitamin E (5,000 mg) did not improve the quality of life or disease progression in ALS patients compared to controls (Graf et al., 2005). Similarly, the use of the antioxidant nanocurcumin (or vitamin E) as an add-on therapy to riluzole in ALS patients did not improve motor function (Graf et al., 2005; Ahmadi et al., 2018). Increased intake of dietary vitamin C and carotenoids, known to be powerful antioxidants, also did not reduce the risk of developing ALS (Fitzgerald et al., 2013). EH301, a combination of two antioxidants; pterostilbene (PT), an analog of resveratrol (found in red wine and berries), and nicotinamide riboside (NR; a form of vitamin B3 promoted as an anti-aging supplement), slows disease progression and improved primary outcomes [ALSFRS-R and forced vital capacity (FVC)] in ALS patients. However, this phase III trial was criticized for its small sample size, short duration, and high rate of patient dropout (De La Rubia et al., 2019). A double-blinded clinical trial, in which oral curcumin (600 mg/day, Brainoil) was administered to ALS patients reduced oxidative stress, and improved aerobic metabolism. These promising results warrant future studies examining the effect of these compounds on motor dysfunction and other primary outcomes in ALS patients (Chico et al., 2018).

An open-label dose-escalation trial found administering another antioxidant and cofactor in the ETC, coenzyme Q10 (CoQ10; Ferrante et al., 2005), resulted in no significant improvement in ALSFRS-R score (Levy et al., 2006). Also, a phase III randomized placebo-controlled trial demonstrated that another compound protective against oxidative stress, dexpramipexole, did not improve motor function in ALS patients (Cudkowicz et al., 2013). Similarly, trials in which a combination of several antioxidants was administered (vitamins C, E, selegiline, selenium, and L-methionine) also failed to show any improvement in ALS patients (Orrell et al., 2008). Therefore, collectively, these studies imply that these antioxidant strategies are not effective in ALS.

Despite the negative outcomes of these studies, edaravone (Radicava), was recently approved by the USFDA (Takei et al., 2017b; Dash et al., 2018). Edaravone slows down the loss of motor function in ALS patients by 33% compared to controls [Writing Group on Behalf of the Edaravone (MCI-186) ALS 19 Study Group, 2017a; Writing Group; Edaravone (MCI-186) ALS 19 Study Group, 2017b; Takei et al., 2017a, b; Bhandari et al., 2018], and inhibits disease progression during the early stages [Writing Group on Behalf of the Edaravone (MCI-186) ALS 19 Study Group, 2017a; Takei et al., 2017b]. A phase II open-label clinical trial demonstrated that edaravone significantly reduced nitrated tyrosine levels in the CSF of ALS patients (Sawada, 2017). Whilst one phase III trial did not reveal a significant difference in ALSFRS-R score when additional patient inclusion criteria were applied [Oakes et al., 2017; Edaravone (MCI-186) ALS 16 Study Group, 2017; Sawada, 2017], another confirmatory phase III placebo-controlled trial showed that edaravone treatment resulted in a statistically significant change in ALS FRS-R primary endpoint over 24 weeks in a subset of ALS patients (Writing Group; Edaravone (MCI-186) ALS 19 Study Group, 2017b; Sawada, 2017; Takei et al., 2017a). However, edaravone causes hypersensitivity and allergic reactions in some patients and may be effective in less than 5% of the ALS population [Writing Group; Edaravone (MCI-186) ALS 19 Study Group, 2017b]. Despite these concerns, edaravone is effective in a subset of ALS patients. Hence, this raises the possibility that other antioxidants and potent modifiers of redox regulation, which have shown promise in pre-clinical models of ALS, should be re-examined in clinical trials (Table 3).

## Pre-Clinical Studies

Most previous preclinical studies have used transgenic mice overexpressing human mutant SOD1. However, these mice do not display the TDP-43 pathology that is present in almost all cases of ALS, and therefore they have been increasingly criticized as a disease model. Urate, also known as uric acid, is a major endogenous antioxidant that is neuroprotective against both astrocyte-induced toxicity in SOD1^G93A^ mice and H_2_O_2_ induced oxidative stress in cells expressing mutant SOD1^G93A^ (Bakshi et al., 2018). Similarly, urate is also neuroprotective by enhancing GSH expression through activation of the Akt/GSK3β/NRF2/GCLC pathway in mutant SOD1^G85R^ cellular and *Drosophila* models (Zhang et al., 2019b). Also, another oxidant, hydrogen sulfide (H_2_S), was effective against mitochondrial dysfunction in SOD1^G93A^ mice by increasing the levels of Ca^2+^ and inhibiting SOD1 aggregation (Pratt et al., 2012; Paul and Snyder, 2018). Therapeutics based on mitochondrial dysfunction and oxidative stress, such as mitochondria-targeted antioxidant [10-(4,5-dimethoxy-2-methyl-3,6-dioxo-1,4-cyclohexadien-1-yl)decyl]triphenylphosphonium methanesulfonate (MitoQ), modify disease progression by slowing the decline of mitochondrial function and disease progression in the SOD1^G93A^ mouse model of ALS (Miquel et al., 2014).

Recent studies have shown that treatment of SOD1^G93A^ mice with naturally occurring flavonoids fisetin and 7,8-dihydroxyflavone, inhibits ROS production and neurodegeneration by activating the ERK signaling pathway (Korkmaz et al., 2014; Wang T. H. et al., 2018). Also, resveratrol improved motor function and survival in SOD1^G93A^ mice through activation of Sirtuin1 (SIRT1) and suppression of oxidative stress (Mancuso et al., 2014; Song et al., 2014). Similarly, epigallocatechin, a naturally occurring antioxidant present in fruits, nuts, and tea, inhibited oxidative stress, induced motor neuron degeneration, and prolonged survival of SOD1^G93A^ mice (Koh et al., 2004, 2006; Xu et al., 2006). Furthermore, lipoic acid, an antioxidant with anti-inflammatory properties, attenuated oxidative stress and neurotoxicity in SOD1^G93A^ and SOD1^G85R^ *Drosophila* and cell culture models (Moura et al., 2015; Wang T. et al., 2018).

NAD^+^, the co-enzyme of reduced NADPH, plays a critical role in redox reactions, energy metabolism, mitochondrial function, calcium homeostasis, DNA repair, and *SIRT1* gene expression (Yaku et al., 2018; Yoshino et al., 2018). However, precursors of NAD+; nicotinamide mononucleotide (NMN), nicotinamide (NAM), nicotinic acid (NA), and NR; are more stable and more easily able to penetrate neurons compared to NADPH, and are protective in SOD1^G93A^ mice and axotomy mice models (Sasaki et al., 2006; Harlan et al., 2016). Enhancing NAD^+^ levels in astrocytes expressing mutant SOD1^G93A^ attenuates toxicity (Harlan et al., 2016). Supplementation of NR activates *SIRT6* expression and delays motor neuron degeneration, reduces neuroinflammation in the spinal cord, and slightly prolongs survival of SOD1^G93A^ mice (Harlan et al., 2016). NR also activates the mitochondrial UPR and prolongs the survival of SOD1^G93A^ mice (Zhou et al., 2020). Furthermore, low doses of the compound diallyl trisulfide, which increases expression of the antioxidant enzymes heme oxygenase1 (HO-1) and NAD(P)H quinone dehydrogenase (NQO1), protects motor neurons from toxicity induced by mutant TDP-43^Q331K^ and TDP-43^M337V^ (Liu C. et al., 2018). Therefore, collectively, these findings suggest that administering NAD^+^ precursors prevents oxidative stress and mitochondrial dysfunction, and hence may be a useful therapeutic target for ALS.

Deletion of NOX by genetic approaches or using NOX inhibitors, is protective against neurotoxicity in ALS disease models (Sorce et al., 2017; Barua et al., 2019). Inactivation of NOX decreases ROS production and prolongs survival in SOD1^G93A^ mice (Seredenina et al., 2016), and may modify survival in ALS patients (Marrali et al., 2014). Furthermore, CPN-9, a novel NRF2 activator, is neuroprotective against mutant SOD1^H46R^ motor deficits and disease progression in mice (Kanno et al., 2012). Also, γ-oryzanol, a mixture of lipids derived from rice, inhibits oxidative stress and neurotoxicity in SOD1^G85R^ *Drosophila* and cell culture models (Zhang et al., 2019a). Several independent studies have also shown that treatment with arimoclomol, a co-inducer of heat shock proteins, is protective against toxic mutant SOD1 aggregates, delays disease progression and improves motor function in SOD1^G93A^ mice (Kieran et al., 2004; Kalmar et al., 2008; Lanka et al., 2009).

CuII [atsm; diacetylbis(4-methylthiosemicarbazonato) copper] is a redox-active molecule involved in redox cycling between oxidized CuII and reduced CuI, that is showing promise as a potential therapeutic in ALS (Hilton et al., 2020). In SOD1^G93A^ mice, CuII (atsm) improves survival by 26% (Hilton et al., 2017) and was therefore more effective than riluzole (3% survival improvement) in this model (Mcallum et al., 2013; Roberts et al., 2014). Moreover, it is the only candidate drug for ALS to be independently validated by the ALS Therapy Development Institute (Soon et al., 2011). A recent Phase I clinical trial found administering CuII (atsm) in ALS patients was safe and well-tolerated (Lincoln, 2018). Phase II trials evaluating the efficacy of CuII (atsm), involving 80 ALS participants, are ongoing.

In summary, at first glance, these studies suggest antioxidant strategies are not effective in ALS, but this may reflect the specific unfavorable pharmacological properties of the antioxidants trialled so far. Harnessing mechanisms that regulate the cellular redox state, rather than the redox molecules themselves, might be more effective in the future. Furthermore, most of the potential redox modifiers described above were only efficacious in SOD1^G93A^ mice, and these mice do not develop the TDP-43 pathology present in almost all ALS cases. Therefore, it will be worthwhile to assess the therapeutic potential of antioxidants and modifiers of redox regulation in TDP-43, C9ORF72, and FUS models of ALS in the future. However, edaravone (Radicava) is one of only two USA-FDA approved treatments currently available for ALS, highlighting the potential importance of restoring redox homeostasis in ALS.

## Discussion/Conclusion

Although links between redox dysregulation and ALS are becoming well documented in the literature, the directionality of these links and their underlying cause is still unclear. However, recent evidence suggests that redox dysregulation may be an important or even primary trigger of a cascade of events leading to neurodegeneration. Notably, TDP-43 pathology is present in almost all ALS cases and specific features can be induced by redox dysregulation; inclusion formation, subcellular localization, and insolubility (Cohen et al., 2012). Reduction of GSH, a vital redox modifier, affects multiple ALS associated proteins. GSH reduction is associated with TDP-43 inclusion formation in sALS patients (Valle and Carrì, 2017) and WT forms of TDP-43 and SOD1 can be induced to misfold and aggregate in neuronal cells (Iguchi et al., 2012; Parakh et al., 2020), thus linking redox dysregulation to protein misfolding and sporadic ALS. Moreover, reduction of GSH also inhibits the protective activity of PDI, which is increasingly implicated in ALS (Parakh et al., 2020).

The clinical manifestations of ALS usually appear between 50–60 years of age, suggesting that neurons die through cumulative damage to normal cellular processes. Given that oxidative stress is associated with aging, prolonged redox dysregulation may therefore induce neurodegeneration, either directly or indirectly (Parakh et al., 2013; Mcbean et al., 2015). Furthermore, redox dysregulation may act as a double-edged sword, inducing a cascade of cellular events that lead to neurodegeneration and simultaneously inactivating protective thiols such as PDI by post-translational modifications. It is therefore imperative to find therapeutics that balance the cellular redox state and regulate redox-regulated proteins associated with key cellular functions.

Redox homeostasis underlies all important cellular activities and ALS is a systemic disease that affects multiple cellular processes. Several studies collectively support the hypothesis that redox dysregulation is central to ALS pathogenesis, particularly in genetically predisposed individuals. Modifiers of redox regulation may therefore be a potential therapeutic target for ALS. Many studies have shown potentially useful effects of antioxidants and modifiers of redox regulation in TDP-43, C9orf72, SOD1, and FUS mice models of ALS. However, previous studies have failed to demonstrate a convincing beneficial effect for redox-active compounds in clinical trials. Therefore, both pre-clinical and clinical studies need to be carefully designed to consider the sample size, primary endpoint, duration of the trial, and animal model used or ALS patient population. It is also possible that the redox modulators previously examined are not the most effective, and additional studies trialling other components of redox regulation may yield more promising findings in the future.

## Author Contributions

CJ wrote the section on direct and indirect evidence for the role of redox dysregulation in ALS, modifiers of redox regulation as a therapeutic target for ALS, contributed to all the figures and tables, and edited the manuscript throughout. SP wrote the introduction, section on cellular redox system, PDI family of proteins, parts of the Discussion, and edited the manuscript throughout. JA conceived the article, contributed additional text, and edited the manuscript throughout for content and style consistency. All authors contributed to the article and approved the submitted version.

## Conflict of Interest

The authors declare that the research was conducted in the absence of any commercial or financial relationships that could be construed as a potential conflict of interest.
